# From Design to Evaluation: Applications of Health Technology Assessment in Myanmar and Lessons for Low or Lower Middle-Income Countries

**DOI:** 10.1017/S0266462319000199

**Published:** 2019-05-17

**Authors:** Saudamini Vishwanath Dabak, Yot Teerawattananon, Thiri Win

**Affiliations:** 1 The Health Intervention and Technology Assessment Program (HITAP), Ministry of Public Health, Thailand; 2 National Health Foundation, Thailand; 3 Saw Swee Hock School of Public Health (SSHSPH), National University of Singapore and; 4 Ministry of Health and Sports, Myanmar

**Keywords:** Health technology assessment, Myanmar, Maternal and child health, Ex-ante evaluation, Ex-post evaluation, Policy process

## Abstract

**Objectives:**

Health technology assessment (HTA) has been widely used to inform coverage decisions in high-income countries over the past few decades and has been getting increasing attention in middle-income countries as a tool for healthcare decision making in recent years. This study aims to use the case of the Maternal and Child Health Voucher Scheme ( MCHVS ) in Myanmar to understand how HTA can have a policy impact in a low or lower middleincome country.

**Methods:**

The stages heuristic framework was used to describe the policy-making process. A document review was conducted and tacit knowledge of researchers involved was recorded. Results. The opportunity for a grant propelled maternal and child health to the policy agenda. An ex-ante HTA, which included a model-based health economic evaluation, informed the design of the scheme. The framework and key parameters from the ex-ante HTA were used for a midaterm review, which provided feedback to the policy implementation process. An ex-post HTA involved fielding a household survey to assess the impact of the scheme.

**Conclusions:**

HTA can be a useful method for informing resource allocation throughout the policy process in low and lower middle-income settings where no formal mechanism for making coverage decisions exists.

Health technology assessment (HTA) is accepted as a tool for setting healthcare priorities to support universal health coverage (UHC) and has been endorsed by member states of the World Health Organization (WHO) (1). HTA has been applied to develop benefits packages and to define lists for pharmaceutical reimbursement, particularly in high-income and upper middle-income countries (2). In addition to promoting the use of evidenceinformed policy, HTA has facilitated the strengthening of the health system infrastructure through the development of national guidelines as well as capacity for conducting HTA research (3).

Few low- and middle-income countries (LMICs) have made strides in using HTA for making decisions on health care. Thailand is among the countries that has applied HTA for developing its non-pharmaceutical and pharmaceutical benefits packages, for the Universal Coverage Scheme (UCS) and National List of Essential Medicines (NLEM), respectively (4;5). In Colombia, an HTA unit emerged through an incremental process, benefitting from multiple “windows of opportunities” (6). Reviews on the appetite for HTA in India, where public expenditure on health is relatively low, show that there are low-hanging fruits that could be tapped into for linking evidence to policy (7;8). There is, however, little documentation of the role of HTA across the spectrum of LMICs where government resources are limited and the need for rationalization of resources is dire.

This study seeks to fill this information gap by demonstrating the application and usefulness of HTA in such countries by describing the case of Myanmar where HTA has been implemented for developing a vertical government program on maternal and child health care. The program, called the Maternal and Child Health Voucher Scheme (MCHVS), was developed in 2010 as part of Gavi, The Vaccine Alliance’s (henceforth, Gavi) Health Systems Strengthening (HSS) support to Myanmar. Through this study, we argue that HTA can be applied throughout the policy process i.e. before, during and after the implementation of a program in countries with limited resources and capacity and that this approach can be used to strengthen the policy process, attract investments in areas that are underfunded and build infrastructure and capacity for health systems research.

## Methods

The principle methods applied for this study were a document review and recording of tacit knowledge of the persons involved in the process. The review covered documents submitted to Gavi as part of the application and monitoring process which are publicly available (9). Reports and articles published in peerreviewed journals on the design and evaluation of the schemes were also assessed (10,11–12). Tacit knowledge is a rich source of information and is drawn on during the process of policy development (13). Hence, to complement the document review, tacit knowledge of the parties involved in the process of developing or implementing the scheme were codified over the course of writing the study. The study was written and reviewed by all three authors who were involved in at least one stage of the process which involved developing or conducting the studies, incorporating their experiences and interpretation of the process.

The stages heuristic framework was used to contextualize the policy process for developing and implementing MCHVS under the Gavi HSS support. Over the decades, scholars have used this framework with variations in the steps included. In this study, we use a simplified version of the framework to cover four key stages: agenda setting, formulation, implementation and evaluation (14). The problem for policy making, which surfaces at the stage of agenda setting, is honed and defined during the formulation stage; the resulting policy is carried out during the implementation stage and finally, assessed for impact at the evaluation stage. This framework provides a useful basis for analyzing the policy process for the purpose of this case study. However, the framework has been criticized for its linear flow of the policy process and its inability to attribute causality or address the role and power of the actors involved (15;16).

The definitions for the different types of HTA are based on those proposed by Teerawattananon et al to categorize HTAs as per their application before, during or after policy implementation (17). An ex-ante HTA relates to the application of technology assessment before choosing or implementing a policy. This type of HTA relies on using existing evidence, often comprising small scale experimental studies, and making assumptions to estimate the likely cost and impact of the proposed policy. Unlike an ex-ante HTA, an on-going HTA can use the data collected during the implementation of the program and focus on context-dependent issues such as adherence to intervention protocols by providers and end users. This on-going HTA should focus on key surrogate outcome indicators identified in the ex-ante analysis. An ex-post HTA is one conducted toward the end of or after the completion of a program or policy. This type of HTA can provide information on the impact of the program or policy by using evidence from implementation and is not based on small scale experimental studies. This HTA framework is consistent with the stages heuristic framework of policy development such that the ex-ante HTA informs agenda setting and formulation, the on-going HTA relates to implementation and the ex-post HTA which is conducted after the implementation period.

## Results

The results of the study are structured around the four stages of the policy process and are described below. We start with the Government of Myanmar’s application for Gavi HSS support in 2008, followed by the ex-ante HTA that led to the design of the scheme, evaluation of early stage implementation and finally, the evaluation.

### Agenda Setting: Need for a Community-based Health Financing Model

Myanmar is a lower middle-income country and spends less than 3 percent of its Gross Domestic Product (GDP) on health and more than 90 percent of total health expenditure comes from out-of-pocket expenditure (OOPE) of households (18). In 2008 , the Government of the Union of Myanmar applied for HSS support under Gavi (9). As part of the proposal development process for the grant that took place over a year, a National Health Sector Coordinating Committee (NHSCC) was set up to coordinate research and stakeholder consultations (19). An analysis of the health system was conducted to identify the barriers to healthcare provision. The dual challenges of service delivery and health financing for maternal and child health received attention in the framework that defined the grant application and was aligned with the National Health Plan. The Millennium Development Goals (MDGs) gave an impetus for achieving key targets on maternal and child health. Furthermore, health financing was prioritized given the low level of public spending and conversely, high level of OOPE. The need for exploring alternative forms of financing health, particularly at the community level, were articulated in the proposal. In this way, a donor priority area, underpinned by analysis, helped bring the plan for a community health insurance scheme to the fore.

### Policy Formulation: Ex-ante HTA

The next step involved transforming the concept of a community health insurance scheme into a program and designing a scheme that would address problems of high maternal and infant mortality ra tes in the country (10). A feasibility study was conducted by the Health Intervention and Technology Assessment Program (HITAP) together with the Ministry of Health, Myanmar, and the World Health Organization (WHO) as described by Kingkaew et al. (20). The principles of HTA were applied to assess the potential impact, value for money and financial feasibility of introducing a maternal voucher scheme. The information derived from this study was discussed at high levels of government, the WHO and the United Nations Children Fund (UNICEF), resulting in the implementation of the scheme in two townships.

To start with, a literature review of demand-side financing schemes was conducted and through consultations with various stakeholders including staff at hospitals, a protocol for developing the community health insurance scheme was developed. The scheme was designed to cover four antenatal care visits, one delivery, and one postnatal care visit. The key features of the scheme are summarized inTable 1.

**Table 1:** Maternal and Child Health Scheme (MCHVS)

What?	The Maternal and Child Health Voucher Scheme ( MCHVS ) covers care provided by skilled healthcare workers for four antenatal care visits, one delivery, one post-natal care, and between three to five visits for immunization of the child.
Who?	Households with low income identified by their income and asset ownership as well as residence in hard to reach areas.
How much?	Monetary incentives are provided to both the beneficiary, pregnant mother, and child, as well as the provider, typically, the mid-wife. The subsidy covered the fee, transportation, and living costs. The voucher covered the services at the health facility and home.

The ex-ante HTA used a model-based economic evaluation to inform decision makers about the impact of introducing MCHVS in a given township in Myanmar. This ex-ante HTA used existing evidence and made assumptions to estimate the likely costs and health impact of the proposed program. Relevant stakeholders were invited to work with HTA experts to ensure the local relevance of the assessment, as well as to strengthen the absorptive capacity of the local authorities, including key staff from the Department of Health Planning, Department of Health, township medical officer as well as local practitioners, by facilitating the consideration of important parameters or factors affecting the potential success of grant implementation.

The results showed that the MCHVS, so designed, had the potential to improve usage of antenatal care from 73 percent up to 93 percent and delivery by skilled birth attendants (SBAs) from 51 percent up to 71 percent (20). The program was also found to be cost-effective with an incremental cost-effectiveness ratio (ICER) of 381,027 kyats or USD 442 per DALY averted, with 2.83 DALYs averted for both mothers and infants (20).

The impact of this ex-ante HTA were manifold. The results of the study were presented to senior decision makers in the Ministry of Health with key staff from the WHO and UNICEF in Myanmar. In March 2011, it was decided that the MCHVS would be implemented in one township commencing in November 2012 (17). In addition to supporting decision making, the HTA study drove local data generation such that key parameters from the ex-ante HTA study were identified by implementation teams and used as a baseline for conducting monitoring and evaluation (M&E). Finally, the study served as a means of building capacity of staff in the Ministry of Health who led the implementation of the scheme (21).

### Policy Implementation: Evaluation of on-going Program Implementation

After seven months of implementing MCHVS in one of the townships, an on-going HTA study was conducted to provide feedback on the implementation process (11). Unlike ex-ante assessments, this on-going HTA focused on context dependent issues such as the willingness of target populations to participate in the program, adherence to intervention protocol by providers and end-users, and response of other sectors to the program through primary research. This HTA focused on the key surrogate outcome indicators identified in the ex-ante assessment. Results from the ex-ante assessment were used as benchmarks for the assessment at this stage.

The findings of the study provided many important recommendations as shown by Pilasant et al. (22). The methods used for this study included interviews, focus group discussions, survey, and analysis of routine data records. This evaluation also adopted many parameters from the first HTA to discern program effectiveness. In terms of the progress in health outcomes, the study found that more than half the eligible women who had been registered for the scheme received the voucher, as required by the protocol for availing services. Notably, usage of target health services increased, especially delivery by SBAs (22).

The study also offered important insights on program implementation (11). It was found that the scheme was promoted in a way that emphasized financial incentives rather than health benefits and reduction in productivity loss. It was, therefore, recommended that the communications strategy highlight health benefits rather than monetary benefits to ensure long-term sustainability of MCH services in Myanmar. Additionally, it was recommended that given the limited human resources, non-midwives, including traditional midwives and community leaders, be enlisted to distribute vouchers to expand the reach of the program to hard-to-reach areas. A third area that emerged from the survey was the lag time of two to three months for reimbursing the costs of services provided against the voucher. A shorter reimbursement window of one week was recommended. The evaluation also showed that in some areas, the vouchers were not adequate in overcoming coverage issues due to lack of professionals and health facilities. This was a barrier to accessing the targeted health services and warranted allocation of staff to such areas. Other recommendations included making beneficiary selection criteria simpler as well as having a data analyst to monitor the program.

### Policy Evaluation: End of Program Evaluation

Equally important as the ex-ante and on-going HTAs is the ex-post HTA. These HTAs can provide information to responsible authorities on programs that have been proven to be effective and good value for money so that these can continue to be financed. Sustainability is a serious concern for all parties involved in the delivery of external aid, and local governments are often confronted with the difficult decision of continuing support for initiatives previously funded by external donors. Ex-post assessments can provide good opportunities to inform decision makers in recipient countries about the usefulness, value for money, and other implications the program might have if it is continued.

In this case, an end-of-program evaluation was conducted for the two schemes under Gavi HSS, the MCHVS, which, by 2016 , had been implemented in one more township, and the Hospital Equity Fund (HEF), which was operating in more than a hundred townships. With a focus on OOPE and equity, a theory of change was conceptualized and seven research questions were developed to assess the impact of the two schemes. Led by the Ministry of Health and Sports (MoHS), with technical assistance from HITAP and support from the WHO, the study used multiple methods: a document review, fielding of a self-assessment form among HEF program managers, analysing of M&E data, and conducting a household survey. The household survey was a major component of the study and involved developing a questionnaire and handbook, translation of tools and training of enumerators. It was also among the early surveys to have used electronic data collection methods. A qualitative study on OOPE undertaken by Save the Children in association with the World Bank Group complements this study.

The chief finding from the implementation of the scheme was about the importance of effective targeting of beneficiaries. The study showed that the MCHVS was effective in reducing catastrophic health expenditure among households that reported using the scheme. The townships where the scheme was implemented were also associated with higher rates of immunization among households (23). However, there were some limitations in the implementation of the scheme given the administrative burden that it placed on the system in terms of voucher management, financial management and human resource management. The MCHVS thus offers one model of reducing financing barriers of households in accessing maternal and child health and can inform the development of the next generation of health insurance schemes in Myanmar.

### Application of the Stages Heuristic Framework to the MCHVS

Given the above, the elements of the stages heuristic framework are summarized for MCHVS implementation in Myanmar in Table 2.

**Table 2:** Summary of the Policy Process for MCHVS

Stages heuristic	Agenda setting	Formulation	Implementation	Evaluation
Type of studies	Situation analysis	Ex-ante HTA	On-going HTA	Ex-post HTA
Year	2008	2010–11	2013	2016–17
Objectives	Setting policy direction, goal(s), and target(s)	− Predict likely costs and impact Assess value for money and financial and program feasibility to inform decision makers to select the best possible option	− Evaluate key parameters to direct more effective program implementation and ensure impact.	− Assess whether the program achieved its goal
Approach	Stakeholder consultation, health system gap analysis	Document review Consultations Evidence synthesis Model-based evaluation	Self-reported questionnaires Collection and analysis of hospital utilization data Focus group discussions and interviews Direct observation	Document review Self-assessment form Analysis of M&E data Household survey
Key results	The need to address issues of high out-of-pocket health expenditures and inability to pay for maternal and child health services provided by skill-birth attendants	Key interventions and their likely cost and value for money determined	Willingness of target populations to participate in the MCHVS, adherence to intervention protocol by providers and end-users	Targeting of beneficiaries is key. Effective in reducing out of pocket expenditure.
Implication for the next phase	Impetus for community health insurance scheme	Key parameters for implementation success developed.	Recommendations to improve implementation	Knowing whether MCHVS should be continued using other financial sources

Note: M&E = Monitoring and Evaluation.

## Discussion and Conclusion

The case study of Myanmar illustrates the feasibility and usefulness of conducting and applying HTA to inform policy formulation, implementation and evaluation in a country without an explicit mechanism for integrating HTA. The Gavi HSS support offered an avenue for reviewing and developing an approach for addressing the service delivery and health financing needs of the country. Various types of HTAs, namely ex-ante, on-going, and ex-post HTAs, then guided the policy formulation, implementation and evaluation of the vertical program for a topic of priority for the government and donor. The end-of program evaluation suggests that the program has been successful and the contribution of HTA is recognizable. To our knowledge, this is the first piece of work delineating the use of HTA throughout the policy process in an LMIC. It benefits from previous work on the topic and integrates the four components of the policy process to enhance the understanding of the role of HTA therein.

The stages heuristic provides a useful framework for this case study but is wanting in some respects. While there is linearity in the process of policy making as shown by this case study, the interplay between the stages of the policy process are evident. For example, problem definition occurs at almost each stage of the process, requiring re-formulation, testing, and implementation. Second, the actors in the policy process play an important role which is not captured by the framework. The decision makers in government, donors, and program officers at the township level, all interacted with the evidence as it was translated into action. These factors call for taking a more political economy approach to the policy development process, which also incorporates the context and processes as proposed by Walt et al. (14).

HTA has typically taken root in high-income countries as indicated by the membership list of the International Network of Agencies for Health Technology Assessment (INAHTA), a global network of HTA agencies (24). As the 2015 WHO Survey on HTA suggests, countries at different levels of income use HTA for different purposes: LMICs typically use HTA for planning and budgeting whereas high-income countries were more likely to use HTA to inform reimbursement of benefits packages (25). However, in this same survey, there was limited participation from low-income countries resulting in over-representation of countries with established HTA systems. This highlights the importance of examining case studies such as the one presented here to understand how HTA can be applied in a country without an established system for the same noting that contextual factors play a significant role in shaping the outcomes.

The study also illustrates the use of HTA for a non-traditional topic, that is, it is not related to pharmaceutical products, medical devices or surgical procedures. Topics such as maternal and child health are often at the top of the agenda of governments in LMICs which are still grappling with issues of primary health care. This case study shows that the topic for HTA needs to be neither “new” nor “sophisticated.” Furthermore, HTA usually functions upstream in the policy process and influences the agenda setting and formulation stages, for example, making coverage decisions of high-cost health technologies. However, in the case of MCHVS, the entire policy cycle has benefitted from an HTA approach to policy making. The increased awareness about HTA has enhanced the appetite for the tool. Going forward, there are opportunities for using HTA for decision making with the setting up of an implementation unit under the National Health Plan for 2017– 21, which is tasked with defining the Essential Package of Health Services among others (26).

Many countries in the South-east Asia region have implemented HTA and a study identified conducive factors such as high level of government expenditure on health care and a solid health information infrastructure that catalyzed their success (27). Two other factors conducive to the use of HTA, political will and effective collaboration with HTA agencies, were observed. Even without legislation, there was support from the polity and there was substantial collaboration with regional entities. Additionally, local stakeholders were involved and cooperated at each step of the process. The infrastructure for health information was built alongside the study whereby data collection forms were later used to record information. Human capacity to conduct such studies has also been built over the course of the program for conducting surveys, for example. On the other hand, some of the conducive factors found elsewhere are not present in Myanmar. This work took place in a context where public health expenditure was not very high, although it has increased over the years. Furthermore, this program was dependent on an international donor and will not continue in its current form.

From the points above, it is clear that building capacity for HTA of partners in countries is critical for instilling confidence and maintaining momentum for evidence-informed policy making. Currently, there are limited entities that provide in-country support to generate evidence for policy making such as the International Decision Support Initiative (iDSI) (28). The possibility of using evidence to inform policy in countries such as Myanmar ought to encourage global donors to find more mechanisms to support this work in LMICs. Regional networks play an important role in this regard and the increased number of members in regional HTA fora such as HTAsiaLink, RedETSA and EUnetHTA are testament to this growing trend. Globally, INAHTA and HTAi provide a platform for knowledge transfer and exchange as well as networking that can help countries develop HTA capacity (29;30).

There are some limitations to the analysis presented in this study. For one, it relies on the experience and interpretation of the authors who have been involved in the studies. Furthermore, the study draws on existing literature although many of these documents have been published in peer-reviewed journals. Regarding definitions, this study does not distinguish between HTA and economic evaluations and the terms are used interchangeably. It may be noted that HTA is a broader concept with a scope that is not limited to the economic aspect of policy implementation but also other factors such as social, political, and ethical factors as costeffectiveness evidence alone has been found to be inadequate for setting healthcare priorities (31). Lastly, there are several approaches and toolkits available to support countries that are implementing HTA in their own setting even though they do not have the necessary infrastructure. For example, Bijlmakers et al. suggest using the EU initiated framework of INTEGRATE framework for HTA for LMICs (32). Moreover, iDSI has proposed a toolkit to develop the process for HTA in countries (33). This study, on the other hand, provides an exposition on the application of HTA in an LMIC setting and, therefore, does not focus on showcasing the functionality of HTA or on developing an HTA process.
